# Plasma Levels of MicroRNA Let-7c-5p May Predict Risk of Acute Chest Syndrome in Patients with Sickle Cell Disease

**DOI:** 10.3390/ijms26083831

**Published:** 2025-04-18

**Authors:** James Fan, Joanna Gemel, Eric C. Beyer, Gabrielle Lapping-Carr

**Affiliations:** Department of Pediatrics, University of Chicago, Chicago, IL 60637, USA; james.fan@uchicagomedicine.org (J.F.);

**Keywords:** sickle cell disease, acute chest syndrome, extracellular vesicles, miRNA

## Abstract

Acute chest syndrome (ACS) is among the most serious complications of sickle cell disease (SCD). While the pathogenesis of ACS is incompletely understood, endothelial damage and microvascular occlusion are critical components. Our previous studies have implicated small extracellular vesicles in the plasma of subjects with SCD in causing endothelial dysfunction. This suggested that microRNAs within these small EVs might be responsible for endothelial damage. The sequencing of microRNAs in small EVs from the plasma of subjects with SCD revealed that several miRNAs were differentially expressed between subjects with and without ACS history, including let-7c-5p. In a replication cohort, plasma let-7c-5p levels were quantified via RT-qPCR. The baseline plasma let-7c-5p level was twofold higher in patients without previous ACS. Furthermore, we observed a positive correlation between let-7c-5p levels and time to subsequent ACS events. These findings suggest a role for let-7c-5p in endothelial disruption underlying ACS pathogenesis. It may also serve as a novel biomarker for ACS detection and the prediction of disease progression.

## 1. Introduction

Sickle cell disease (SCD) is a common monogenic disorder that affects millions of people worldwide. In SCD, a single amino acid substitution (Glu6→Val in β-globin) produces an abnormal hemoglobin that polymerizes when deoxygenated, resulting in sickled red cells. The sickled erythrocytes are rigid and abnormally shaped, and they cause the intermittent occlusion of small blood vessels. Hemolysis, repetitive ischemia/reperfusion injuries, and the subsequent host immune response cause localized and persistent endothelial damage and may produce damage in many different organs [1,2]. Endothelial damage is central to the pathology of SCD and its crises, including acute chest syndrome (ACS) [3].

ACS is a major cause of premature death in children (and adults) with SCD. It is a disorder characterized by pulmonary inflammation and endothelial damage. Episodes of ACS can be triggered by viral infections, bacterial infections, and ischemia from obstruction or fat embolism [3]. While there is a general consensus regarding diagnosis and supportive care for patients with ACS, many pathophysiologic details remain unclear and clinical care could be improved [4,5]. A better understanding of molecular aspects of ACS pathophysiology might lead to improvements in treatments and might perhaps prevent ACS episodes from occurring. The discovery of a biomarker would allow for the prediction, risk stratification, and potential prevention of ACS [6].

In recent studies, we have been focusing on the possible involvement of small extracellular vesicles (small EVs, sometimes called exosomes) in the pathogenesis of SCD complications. Extracellular vesicles (of different sizes) are contained in the plasma and have been implicated in various vascular diseases (including SCD), since they can modulate endothelial dysfunction and damage, ischemia/reperfusion injury, thrombosis, and inflammation (reviewed by [7,8,9,10]). We found an increased abundance of small EVs in the plasma of children and young adults with SCD [11,12]. These EVs have several characteristics matching those of exosomes, including similar size (~100 nm diameter) and the presence of typical exosomal proteins (CD63 and flotillin-1) [13]. We demonstrated that SCD EVs cause the disruption of intact monolayers of cultured endothelial cells in a variety of different ways: extracellular impedance is reduced, spaces open between cells, the abundance of adherens junctions containing VE-cadherin is reduced, and these junctions are disrupted [11,12,13]. SCD EVs also disrupt other classes of intercellular junctions containing ZO-1 or connexin43 [12,14]. If EVs are obtained during an episode of ACS, they are found to cause more severe endothelial disruption than EVs obtained from the same subject when at a baseline good state of health [13]. Similar studies showed increased endothelial disruption from EVs obtained during a vaso-occlusive episode compared to baseline samples obtained from the same subject [15].

MicroRNAs are contained within small EVs and have been implicated as responsible for many of the effects of small EVs, especially ones that do not occur immediately and involve changes in gene expression. Therefore, in the present study, we determined the microRNA contents of small EVs isolated from the plasma of subjects with sickle cell disease and the relation between levels and a patient’s history of ACS.

## 2. Results

### 2.1. Characteristics of Subjects Used for RNA Sequencing

We queried an SCD biobank and identified 29 subjects with blood samples obtained at baseline, including 12 with no history of ACS (ACS−) and 17 with a history of ACS (ACS+).

In Table 1, demographic data, clinical characteristics, and some hematological laboratory values for the subjects with SCD are shown. The median patient age was 14 years in both groups, and the groups did not differ in the proportion of males and females. Most patients had the hemoglobin SS genotype. Both groups included one patient with the SC genotype, and the ACS+ group contained one subject with the Sβ^0^Thal genotype.

Most hematologic values did not differ between the two groups. While the absolute number of ACS episodes and the rate of ACS episodes were very different between groups, the two groups included similar proportions of subjects with a history of other SCD-related clinical events (pain, cholecystectomy, splenectomy). The proportion of patients receiving hydroxyurea was significantly higher in the ACS+ group and likely explains the higher MCV values in this group. The ACS+ group also contained more subjects carrying a diagnosis of asthma.

### 2.2. MicroRNA Contents of Small EVs in Subjects with SCD

**Figure 1:**
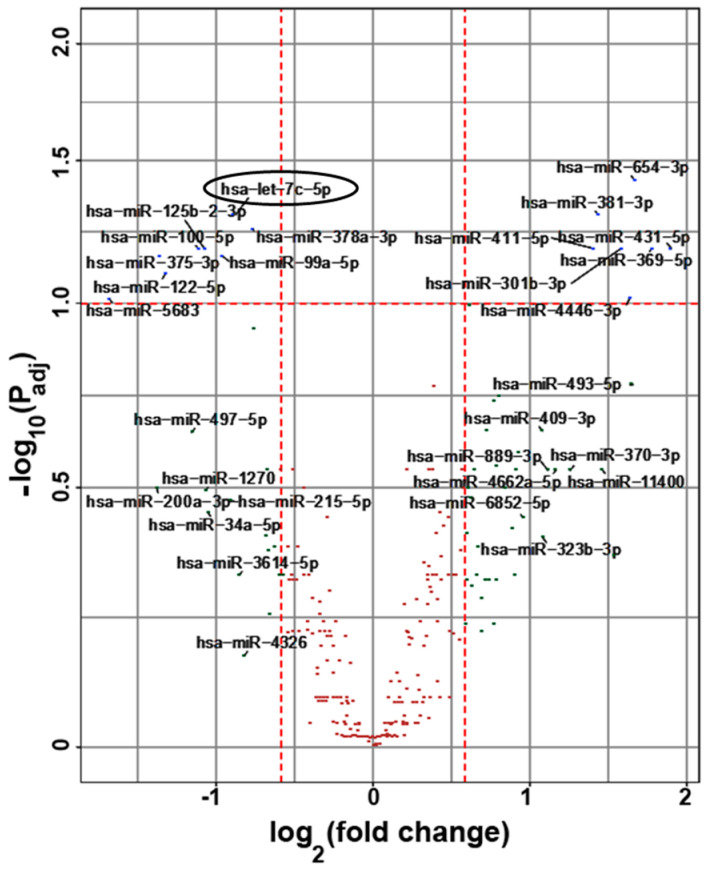
Correlation of plasma small EV levels of different miRNAs with history of ACS episodes in subjects with SCD. The graph shows a Volcano plot of miRNAs identified in small EVs from subjects with SCD at baseline. Small EVs were isolated from subjects with SCD that either had a history of ACS (*n* = 17) or did not (*n* = 12). miRNAs isolated from the EVs were sequenced, and expression was analyzed using DESeq2. The upper outer quadrants (corresponding to fold change ≥ 1.5 and false discovery rate < 0.1) contain several (15) miRNAs that are significantly differentially expressed based on a history of ACS, including mir-let-7c-5p, which is enclosed by a circle. One dotted vertical red line is drawn at log_2_ (fold change) = −0.58, which corresponds to 1.5-fold downregulation. All miRNAs to the left of this line are downregulated at least 1.5-fold. Another dotted vertical red line is drawn at log_2_ (fold change) = 0.58, which corresponds to 1.5-fold upregulation. All miRNAs to the right of this line are downregulated at least 1.5-fold. The dotted horizontal red line at −log_10_ (P_adj_) = 1 corresponds to a false discovery rate of 0.1. All miRNAs above this line have a false discovery rate lower than 0.1.

Small EVs were isolated from the plasma of the subjects with SCD. RNA (enriched for small RNAs, including microRNAs) was isolated from the EVs. This RNA was subjected to sequencing at the Genomics Facility of the University of Chicago. Comparisons showed that several (~15) microRNAs were differentially expressed between subjects with vs. without a history of prior ACS (Figure 1). Among these microRNAs, let-7c-5p was the one with the most significant increase in ACS+ subjects vs. ACS− subjects.

### 2.3. Characteristics of Subjects Used for RT-qPCR of Let-7c-5p (Replication Cohort)

We identified a second group of 16 subjects (from the SCD biobank) with blood samples obtained at baseline, including 6 with no history of ACS (ACS−) and 10 with a history of ACS (ACS+). In Table 2, we present demographic data, clinical characteristics, and some hematological laboratory values for these subjects. The median patient ages in these groups were slightly younger than in our initial group. The groups did not differ in the proportion of males and females. All subjects had the hemoglobin SS genotype, except one subject in the ACS− group who had the Sβ^0^Thal genotype. The proportion of patients receiving hydroxyurea was significantly higher in the ACS+ group, as was the diagnosis of obstructive sleep apnea. The groups did not differ significantly in the prevalence of asthma diagnoses.

### 2.4. Let-7c-5p Levels in Subjects with SCD

We sought to quantify any differences in the expression of microRNAs in this “replication” cohort.

**Figure 2:**
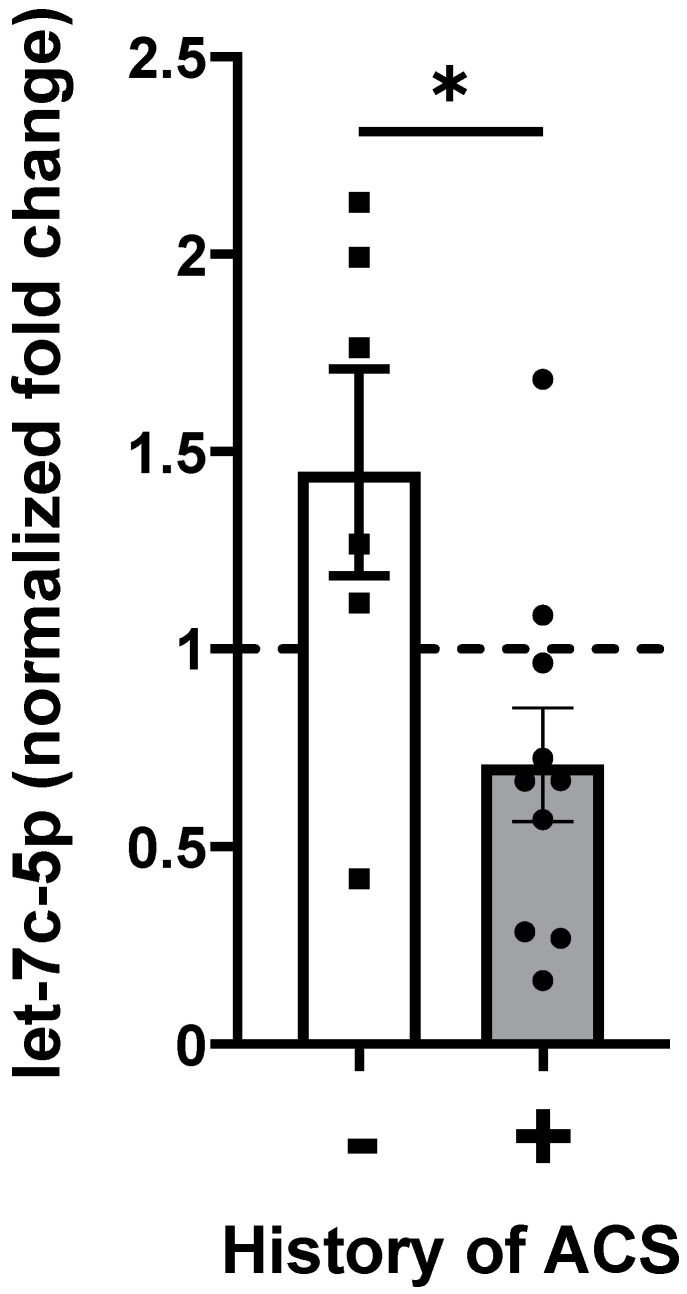
Relation of plasma levels of let-7c-5p miRNA to history of ACS in subjects with SCD. RNA was isolated from baseline plasma samples obtained from an independent set of subjects with a history of ACS (*n* = 10) and from subjects without a history of ACS (*n* = 6). Let-7c-5p levels were quantified by real-time RT-qPCR. Results were normalized to the value of control subjects. * indicates *p* < 0.05 (unpaired *t*-test). Error bars indicate ± standard error of the mean. Circles and squares indicate individual subject data points. Dashed line indicates 1.0.

Total RNA was isolated from platelet-free plasma from the 16 patients and quantified using RT-qPCR. Initially, we chose a few microRNAs that were differentially expressed between subjects with vs. without a history of prior ACS in the discovery cohort for further analysis in the replication cohort. These included let-7c-5p, miR-375-3p, miR-100-5p, and miR-122-5p. Our analyses showed that let-7c-5p was adequately abundant and could be most reliably quantified in all samples, leading us to focus further studies on let-7c-5p. We found that levels of let-7c-5p were on average significantly higher in subjects with no history of ACS compared to patients with a history of ACS (Figure 2).

**Figure 3:**
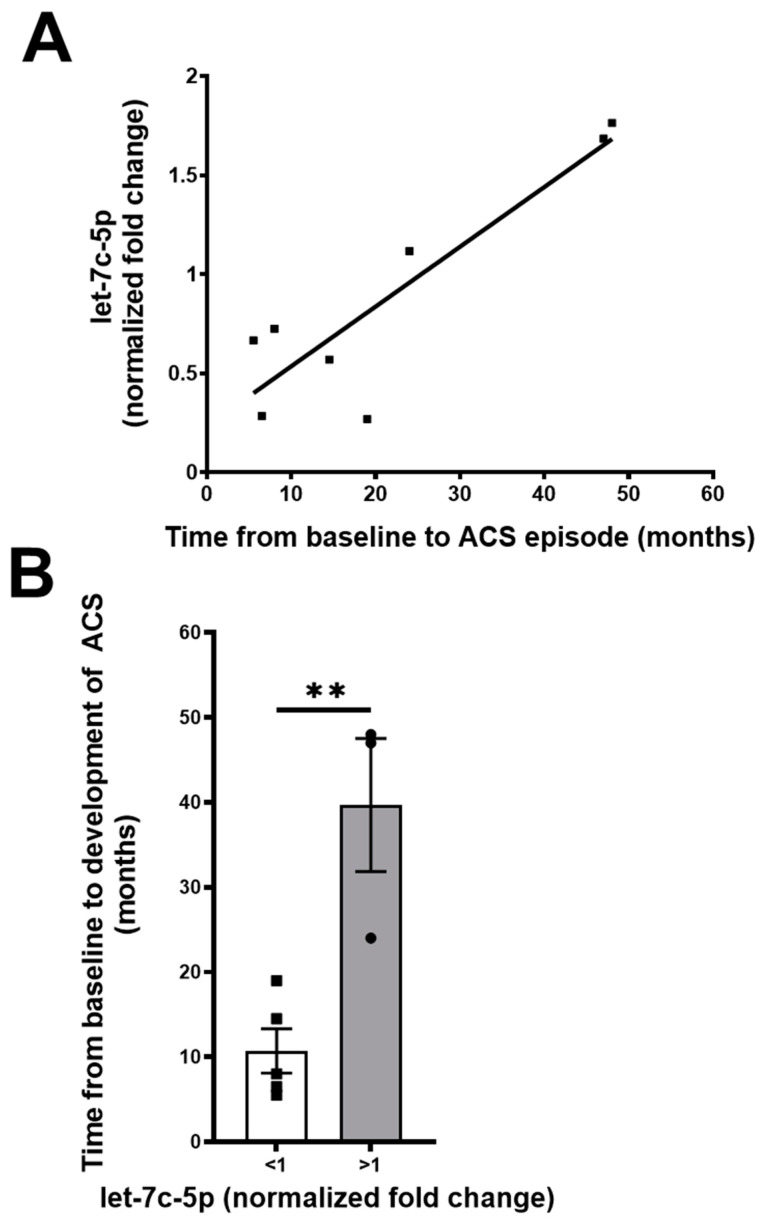
Relationship between let-7c-5p levels and time to the development of ACS. (**A**) Graph depicts let-7c-5p values in comparison with the time to the development of a subsequent or initial ACS episode. Values are best fit by y = 0.03x + 0.2; R^2^ = 0.8; *p* = 0.003. (**B**) Bar graph compares the times to the development of ACS between subjects with levels of miR-let-7c-5p that are less than or greater than 1 (normalized to the value of control subjects. ** indicates *p* < 0.005 (unpaired *t*-test). Circles and squares indicate individual subject data points. Error bars indicate ± standard error of the mean.

During the course of our sample collection, several of the subjects in both groups (two in the ACS− group and six in the ACS+ group) developed new episodes of ACS. Therefore, we analyzed the relation between the values of baseline let-7c-5p levels and the time to the development of ACS. We found a significant correlation: patients with higher baseline let-7c-5p levels took longer to develop ACS than those with lower levels (Figure 3A). Indeed, we found that subjects with a normalized let-7c-5p value (fold change) of less than or greater than 1.0 differed significantly in the time to the development of ACS between 10 and 40 months (Figure 3B).

## 3. Discussion

Our previous work implicated small EVs (in the plasma of patients with SCD) in the pathogenesis of endothelial damage and ACS. The present study extends that work. We found that these small EVs contain several microRNAs. Among the vesicular microRNAs, the levels of let-7c-5p are higher in subjects with no history of ACS than in subjects with a past history of ACS. We also found a strong positive correlation between the quantity of miR-let-7c at baseline and the time to the development of an ACS episode. A minor limitation of our study is the relatively small numbers of patients included in the two groups. However, this is a common issue with this type of research, and we believe that our subjects are representative of the population.

Our findings suggest that let-7c-5p might serve as a protective factor against the subsequent development of ACS. This hypothesis is consistent with current knowledge regarding this microRNA. Let-7c-5p is a member of the let-7 family of microRNAs [16]. Let-7 was originally identified when microRNAs were discovered in *C. elegans* [17]. The human let-7 family contains multiple different members with small nucleotide differences between isoforms [18]. In many different organisms and cell types, let-7 suppresses cell proliferation and promotes differentiation by suppressing the translation of proteins including MYC, RAS, cyclin D, and HMGA_2_ [16]. The binding of Lin-28 blocks the processing of the precursor form of let-7, thereby inhibiting its actions [19,20]. The let-7/Lin-28 system may have important influences upon the growth and differentiation of various stem cells and cancers [18,19]. By modulating antibody and cytokine production and macrophage function, let-7 has been implicated in immunity and responses to viral infections (reviewed in [21,22]). While there have been relatively few studies that have specifically considered let-7c-5p, several have produced findings that are relevant to our current study. Let-7c-5p has been found in plasma exosomes in different studies [23,24]. Let-7c-5p has been implicated in the regulation of several proinflammatory cytokines [24,25]. Roles for let-7c-5p in vascular function have been supported by data implicating it in cerebral ischemia injury, stable coronary artery disease, and essential hypertension [23,25,26,27].

Our findings also suggest that let-7c-5p might be a useful biomarker to predict the development of ACS. The availability of reliable biomarkers might help predict which patients will develop ACS, and it might lead to earlier detection of ACS, facilitating prompt intervention and management. There have been previous studies evaluating potential biomarkers for ACS, including secretory phospholipase A2 (sPLA2), endothelin-1, interleukin-6, and peripheral white blood cell count [28]. In initial studies, sPLA2 levels were shown to be elevated in hospitalized patients with ACS or with incipient ACS [28,29]. A subsequent review and meta-analysis of multiple studies confirmed a significant association between elevated sPLA2 levels and increased ACS risk in patients with SCD, but the sensitivity and specificity of this marker were not great [30]. Several studies have shown that the endothelial vasoactive mediator endothelin-1 is increased in ACS [31,32]; moreover, germline variants of endothelin-1 and endothelial nitric oxide synthase may put patients at increased risk for ACS [33,34]. Other potentially useful biomarkers of vascular damage or inflammation include soluble Intercellular Adhesion Molecule-1, Angiopoetin-2, and interleukin-6 [35].

## 4. Materials and Methods

### 4.1. Subjects

Subjects with SCD were selected from a registered biobank of patients with SCD from University of Chicago Comer Children’s Hospital and LaRabida Children’s Hospital. Subjects in the biobank were prospectively enrolled. Informed consent was provided by parents or by patients aged >18 years. Assent was obtained from subjects aged 9–18 years. Subjects under 2 years of age were excluded. Baseline samples were drawn from subjects who had been in a steady state of disease (free of infection, new pain, or transfusion) for at least 4 weeks. ACS was defined as a new infiltrate on a chest X-ray accompanied by the requirement for the delivery of supplemental oxygen, tachypnea, wheezing, cough, or chest pain. The rate of ACS was calculated by dividing the total number of events by the number of patient-years at the time of the blood draw. Subjects were defined as having asthma if they had documentation of an International Classification of Disease code for asthma (in their electronic medical record) or if they were prescribed a long-acting asthma controller medication. Procedure codes were used to identify splenectomy and cholecystectomy. Obstructive sleep apnea was diagnosed by a sleep study.

As in our previous studies [13,15], the RT-qPCR experiments included control subjects (without sickle cell disease). For the current study, the control subjects included two patients who were having blood drawn for screening purposes or testing for iron deficiency. Neither of the control subjects had obesity, asthma, or another inflammatory disorder.

All protocols were approved by the Institutional Review Board (protocol # 14-0466 and 15-0263) and were conducted in accordance with the guidelines set by the Declaration of Helsinki.

### 4.2. Isolation of Platelet-Free Plasma

Professional phlebotomists collected blood (~6 mL) from the subjects into EDTA-containing (lavender top) tubes. The blood was centrifuged at 2500× *g* at 24 °C for 15 min, and platelet-free plasma was removed (without disturbing the buffy coat). Then, the plasma was recentrifuged, and frozen (in aliquots) at −80 °C. When needed for study, samples were thawed and recentrifuged at 3000× *g* for 20 min at room temperature prior to the isolation of EVs.

### 4.3. Small RNA Isolation and Its Sequencing in Discovery Cohort

EVs were isolated from platelet-free plasma using the Total Exosome Isolation kit (Thermo Fisher Scientific Inc., Waltham, MA, USA). For each isolation, 400 µL of plasma was used. Pellets containing EVs were resuspended in Qiazol. RNA (including small RNAs) was purified with the miRNeasy Micro Kit (Qiagen, Germantown, MD, USA), resulting in 14 µL of RNA in H_2_O. The concentration of RNA was assessed using an Agilent 2100 Bioanalyzer (Agilent Technologies, Inc., Santa Clara, CA, USA); the average concentration was 250 pg/µL. Libraries were prepared using the Illumina small RNA library preparation kit (Illumina Inc., San Diego, CA, USA). Gel extraction was performed for sample purification, and single-read 50 (SR50) base sequencing was performed using a HiSeq4000 sequencer (Illumina Inc., San Diego, CA, USA) at the Genomics Facility of the University of Chicago.

### 4.4. Sequencing Data Analysis

The data were analyzed by the Center for Research Informatics of the University of Chicago.

The quality of raw sequencing data was assessed using FastQC [36]. The sequences of Illumina adapters/primers were detected from sequencing reads. All RNA reads were initially mapped to the human (hg38) reference genome using STAR release with default parameters [37]. Picard was used to collect mapping metrics [38].

The files resulting from the alignment step were taken individually as input to evaluate transcriptional expression using subread::featureCounts [39]. The differential expression analysis tool DESeq_2_ Release (3.20) was employed to discover differentially expressed genes between pairwise groups based on an expression estimation of individual genes [40]. Differentially expressed miRNAs were discovered through a gene-wise statistical test based on quantified miRNA genes. The criteria used included fold change ≥ 1.5 and false discovery rate (FDR) < 0.1 [41].

### 4.5. MicroRNA Extraction and RT-qPCR in Replication Cohort

Total RNA, including small RNAs, was isolated from 200 µL patient platelet-free plasma using the miRNeasy Serum/Plasma Kit (Qiagen, Germantown, MD, USA) according to the manufacturer’s protocol with a few additional steps. Specifically, platelet-free plasma was centrifuged at 16,000× *g* at 4 °C for 10 min, and the supernatant from this step was used for RNA purification. Prior to extraction with chloroform, 20 µg glycogen (SIGMA-Aldrich, Saint Louis, MO, USA) was added as a carrier/co-precipitant. We also added 6.6 fmol of an exogenous microRNA from *C. elegans*, cel-miR39 (Qiagen, Germantown, MD, USA), as an exogenous spike-in control to monitor the efficiency of RNA extraction [42]. Lastly, the column was incubated for 1 min with eluent (water) prior to the final spin/recovery of RNA. The concentration of RNA was assessed using an Agilent 2100 Bioanalyzer (Agilent Technologies, Inc., Santa Clara, CA, USA); RNA yields were 230–750 pg/µL.

The extracted RNA was reverse-transcribed using the TaqMan Advanced miRNA cDNA Synthesis Kit (Thermo Fisher Scientific Inc., Waltham, MA, USA). PCR amplification was conducted using microAmp Fast optical 96-well reaction plates and the 7500 Fast Real-Time PCR System (Applied Biosystems, Waltham, MA, USA). Assays were performed using preformulated primers and PCR conditions, using assay 478577 for let-7c-5p and assay 478293 for cel-miR-39-3p (TaqMan^TM^ Advanced miRNA Assay, Thermo Fisher Scientific Inc., Waltham, MA, USA). Primer sets were only considered acceptable if their efficiencies were within a 95% confidence value and they did not produce primer dimers (as assessed by the melting curves). All reaction mixes contained RNA derived from equal amounts of plasma. Each sample was run in triplicate. Each plate contained the two control samples from healthy patients. Due to the lack of reliable housekeeping miRNAs, we used the cel-miR39 “spike-in” control. Data were initially normalized to cel-miR39, and then the relative expression of let-7c-5p was calculated using the ΔΔCT method; all values were normalized to the average of the two control subjects.

### 4.6. Statistical Analyses

All statistical analyses of the patient characteristics or of the RT-qPCR data were conducted using GraphPad Prism 10 (GraphPad Software, Boston, MA, USA) applying Chi-square, Fisher’s exact, and unpaired *t*-tests as appropriate. A *p*-value < 0.05 was considered significant for all analyses.

## 5. Conclusions

In summary, our new data show that let-7c-5p is contained in plasma exosomes from subjects with SCD, and its levels correlate inversely with the development of ACS. Thus, let-7c-5p represents a novel molecule for study as a protective factor and biomarker for the development of ACS.

## Figures and Tables

**Table 1:** Patient characteristics of subjects with sickle cell disease used for sequencing small RNAs contained in plasma extracellular vesicles.

	No History of ACS (*n* = 12)	History of ACS (*n* = 17)	*p*-Value
**Demographics**			
Age (years), median (range)	14 (3–18)	14 (5–20)	NS
Sex, n (%)			NS
Male	7 (58)	11 (65)	
Female	5 (42)	6 (35)	
**Hematologic Values**	Median (25%, 75%)	Median (25%, 75%)	
White blood cell count (×10^3^/µL)	9.2 (8.4, 14.3)	11.8 (10.7, 14.6)	NS
Hemoglobin (g/dL)	8.4 (8.1, 9.1)	9.3 (7.8, 10.7)	NS
MCV (fL)	84.2 (77.3, 93.8)	95.2 (88.3, 101)	<0.05
Reticulocyte (×10^3^/µL)	266 (212, 384)	249(161, 303)	NS
Platelet (×10^3^/µL)	404 (291, 462)	414 (400, 458)	NS
Bilirubin (mg/dL)	3.0 (2.0, 4.6)	2.9 (1.8, 6.3)	NS
**Hemoglobin Genotype, n (%)**			
SS	11 (92)	15 (88)	
Sβ^0^Thal	0	1 (6)	
SC	1 (8)	1 (6)	
**Clinical Characteristics**			
Rate of ACS (n/year)	0	0.17 (0.08, 0.23)	<0.05
Absolute ACS, n	0	1 (1, 3.5)	<0.005
Rate of pain (n/year)	0 (0, 0.23)	0.18 (0.08, 0.65)	NS
Absolute pain, n	0 (0, 3.5)	2 (1, 9)	NS
Hydroxyurea, n (%)	3 (25)	12 (71)	<0.05
Asthma, n (%)	0	5 (29)	<0.05
Obstructive sleep apnea (%)	6 (50)	4 (24)	NS
Splenectomy, n (%)	3 (25)	4 (24)	NS
Cholecystectomy, n (%)	1 (8)	4 (24)	NS

**Table 2:** Patient characteristics of subjects with sickle cell disease used for qRT-PCR studies of let-7c-5p.

	No History of ACS (*n* = 6)	History of ACS (*n* = 10)	*p*-Value
**Demographics**			
Age (years), median (range)	4 (4–8)	8 (4–9)	NS
Sex, n (%)			NS
Male	3 (50)	3 (30)	
Female	3 (50)	7 (70)	
**Hematologic Values**	Median (25%, 75%)	Median (25%, 75%)	
White blood cell count (×10^3^/µL)	12.3 (10.6, 13.2)	10.9 (9.9, 12.4)	NS
Hemoglobin (g/dL)	8.3 (8.2, 8.7)	9.2 (8.0, 9.7)	NS
MCV (fL)	88.6 (84.1, 92.4)	93.5 (91.4, 98.0)	NS
Reticulocyte (×10^3^/µL)	258 (217, 298)	231 (203, 259)	NS
Platelet (×10^3^/µL)	402 (282, 509)	429 (383, 459)	NS
Bilirubin (mg/dL)	1.9 (1.9, 2.6)	3.1 (2.1, 4.7)	NS
**Hemoglobin Genotype, n (%)**			
SS	5 (83)	10 (100)	
Sβ^0^Thal	1 (14)	0 (0)	
**Clinical Characteristics**			
Rate of ACS (n/year)	0	0.25 (0.20, 0.25)	<0.005
Absolute ACS, n	0	1.00 (1.00, 2.75)	<0.005
Rate of pain (n/year)	0.6 (0, 0.2)	0.5 (0.1, 0.8)	NS
Absolute pain, n	0.5 (0, 1.0)	2.0 (1.0, 7.0)	NS
Hydroxyurea, n (%)	1 (12)	6 (60)	<0.05
Asthma, n (%)	1 (12)	3 (30)	NS
Obstructive sleep apnea (%)	0	4 (40)	<0.05
Splenectomy, n (%)	1 (12)	2 (20)	NS
Cholecystectomy, n (%)	0	1 (10)	NS

## Data Availability

miRNA sequencing data have been deposited in the Gene Expression Omnibus (GEO) with Accession Number GSE291666 and are openly available. All other data are completely included in the manuscript.

## Abbreviations

The following abbreviations are used in this manuscript:

SCD	Sickle cell disease
ACS	Acute chest syndrome
RT-qPCR	Quantitative reverse transcription polymerase chain reaction
EV	Extracellular vesicle
NS	Not Significant
sPLA2	Secretory phospholipase A2
